# How can we measure the developmental impacts of the COVID-19 pandemic on young children?

**DOI:** 10.23889/ijpds.v7i3.1806

**Published:** 2022-08-25

**Authors:** Mitana Purayastha, Stephen Roberts, Julian Gardiner, Daniel Brison, Scott Nelson, Deborah Lawlor, Alastair Sutcliffe

**Affiliations:** 1 University College London; 2 University of Manchester; 3 University of Glasgow; 4 University of Bristol

## Objectives

To establish a cohort of children consisting of those born after assisted reproductive technology (ART) in the UK between 1992 and 2009, their naturally conceived siblings (NCS) and matched naturally conceived population controls (NCP) and linking this to postnatal health records up to 2015.

## Approach

Deterministic record linkage between the Human Fertilization & Embryology Authority (HFEA) register and Office for National Statistics (ONS) birth registration datasets was carried out to identify a cohort of children born after ART between 1992 and 2009, their NCS, and matched NCP controls (HFEA-ONS linkage). This cohort was linked to the UK Hospital Episode Statistics database to allow monitoring of the child’s post-natal health outcomes up to 2015 (HFEA-ONS-HES linkage). Birthweight and health outcomes were compared between groups and by treatment type.

## Results

The HFEA-ONS linkage consisted of 75348 children born after non-donor ART carried out in the UK between 1st April 1992 and 31st July 2009, 14763 NCS and 164823 matched NCP controls. Of these, 63877 ART, 11343 NCS, and 127544 matched NCP controls were linked to hospital admissions and outpatient data (HFEA-ONS-HES sub-cohort). The study had 1.6-million-person years of follow-up (mean: 12.9 years; range 0-19 years). Children born after fresh embryo transfers were lighter and those born after frozen embryo transfers were heavier than the NCP controls. The within-sibling analyses were directionally consistent with the NCP analyses, but attenuated for the fresh vs NC and increased for the frozen vs NC analyses. ART singletons had increased risk of hospital admission as well as higher admission rates compared to NCP but not NCS.

## Conclusion

Bespoke record linkage was carried out to generate a new child cohort for use in exploring the relationship between conception via ART and short- and long-term health outcomes in offspring. Identification of NCS as well as matched NCP controls allows exploration of the association of ART with adverse offspring outcomes while accounting for parental factors related to sub-fertility which may confound these associations.

